# Investigation of Ratiometric Indices for Determining the Technological History of Dairy Systems Using Raman Spectroscopy

**DOI:** 10.3390/molecules31213890

**Published:** 2026-10-28

**Authors:** Svetlana E. Dimitrieva, Andrey N. Lobanov, Sergey N. Nikolaev

**Affiliations:** https://ror.org/01jkd3546 P.N. Lebedev Physical Institute of the Russian Academy of Sciences, Leninskiy Prospekt 53, Moscow 119991, Russia; dimitrievase@lebedev.ru (S.E.D.); nikolaevsn@lebedev.ru (S.N.N.)

**Keywords:** Raman spectroscopy, reconstituted milk, milk adulteration, ratiometric analysis, principal component analysis, chemometrics, explainable AI (XAI), spray drying

## Abstract

Determination of the history of food processing, specifically the detection of undeclared use of reconstituted ingredients instead of fresh ones, remains a serious challenge. In dairy systems, such detection is difficult because conventional chemical markers reflect the cumulative history of time and temperature changes rather than specific processing steps. Although Raman spectroscopy is widely recognized as a non-destructive, high-throughput tool for screening molecular alterations, physicochemically interpretable ratiometric indices are still required, particularly for use in explainable AI models. To identify diagnostic spectral variances, a single batch of raw whole milk was evaluated across distinct states: raw, pasteurized (76, 88, and 98 °C), and spray-dried at outlet air temperatures of ≤70 °C and ~95 °C. The resulting whole milk powders were reconstituted to a 12.1% total solids mass fraction. Raman spectra were acquired using an M532 microscope (λ = 532 nm), and the analytical band areas at 1005, 1063, 1083, and 1121 cm^−1^ were identified as key diagnostic markers. The structural indices *I_lac_* and *I_prot_*, which are mathematically orthogonal within the principal component space, were subsequently established. As a preliminary proof-of-concept, a binary logistic regression model achieved complete geometric separation between non-dried milk (including high-temperature pasteurized samples) and reconstituted milk. Within the training dataset, the false-positive rate for non-dried milk did not exceed 3.2%, whereas reconstituted milk classification model-predicted probability of belonging to the reconstituted class ranged from 96.8% to 99.9%. Furthermore, the proposed indices show great promise for classifying milk powders based on their heat treatment level. Nevertheless, further validation on independent commercial batches is strictly necessary to evaluate the proposed method’s robustness against matrix and instrumental variability.

## 1. Introduction

In the dairy industry, the unauthorized use of reconstituted dry milk ingredients remains a form of fluid milk adulteration that is difficult to detect [1,2,3,4]. Economically motivated adulteration of dairy products remains a significant issue: the use of cheaper raw materials lowers production costs, it also misrepresents the product’s manufacturing method [4]. Consequently, developing analytical methods to detect the use of dry milk is a priority for authenticity control.

Furosine, lactulose, and other compounds formed via the Maillard reaction and lactose isomerization are used to assess the intensity of heat treatment applied to milk. The utility of these indicators for characterizing the thermal history of dairy products has been reviewed by van den Oever and Mayer [5], while Ho et al. [6] utilized the simultaneous determination of furosine and lactulose to distinguish between fresh and reconstituted skimmed milk. However, the levels of these compounds are influenced by the cumulative time–temperature exposure; intensive pasteurization, sterilization, concentration, and drying can induce similar changes, thereby limiting the specificity of individual markers [7]. Digital colorimetry combined with infrared spectroscopy [8] and tristimulus colorimetry [9] have been proposed as alternatives; however, the robustness of the detected parameters against variations in product composition and processing history requires further evaluation.

Spray drying and the subsequent reconstitution of milk involve not only thermal exposure but also transitions between different states of the dispersed system. Water removal alters the hydration conditions of macro-components, leads to the denaturation and aggregation of whey proteins, rearranges casein micelle interactions, and shapes the structure of the dry product particles [10,11]. The nature of these transformations is determined by a combination of processing parameters [12], while prior heat treatment influences the technological properties of the milk powder [13]. Upon reconstitution, the mass fractions of major components may approximate their initial levels, whereas the colloidal organization and intermolecular interactions do not necessarily revert to the state of the native milk system. Therefore, it is advisable to assess reconstitution not only according to the concentration of individual substances but also the relative response of structurally sensitive spectral regions.

Raman spectroscopy offers a promising approach to this task, enabling the analysis of aqueous dispersions without chemical sample preparation and providing information on the molecular vibrations of proteins, carbohydrates, and lipids. Li-Chan [14] has systematized the application of vibrational spectroscopy to milk proteins, while Silva et al. [15] have summarized the potential of Raman spectroscopy for analyzing the composition, quality, and authenticity of dairy products. Reiner et al. [16] demonstrated the sensitivity of spectra to changes occurring during the production of drinking milk, and Barkovskaya et al. [17] identified temperature-induced changes in the spectral regions of pasteurized milk. Hussain Khan et al. [18] demonstrated the feasibility of determining fat, protein, and lactose content in raw milk flow, positioning the method as a potential component of process analytical technology. However, traditional end-product chemical markers are inherently limited as they integrate the cumulative thermal load rather than distinguishing specific technological transitions, such as the phase shifts occurring during spray drying. To overcome this limitation, analytical focus must shift from bulk concentrations to the underlying macromolecular conformation and structural organization of the dairy matrix. In this context, evaluating the precise steric rearrangements of proteins and the state of carbohydrate crystallization offers a more robust framework for process-specific authentication. Raman spectroscopy enables the real-time monitoring of subtle conformational rearrangements in protein macromolecules, phase transitions in carbohydrates, and fluctuations within the lipid pool [19,20]. A significant portion of research on dairy product identification relies on analyzing the full spectral dataset using principal component analysis, partial least squares discriminant analysis, and machine learning algorithms. Multivariate models have been employed to assess the quality and authenticity of milk powder [19,21], while modern algorithms allow for the classification of dairy product groups based on spectral features [22,23]. However, the transferability of such models depends on the representativeness of the training set and the outcomes of external validation. Ní Fhuaráin et al. [24] have highlighted the necessity of further verifying spectroscopic calibrations prior to industrial implementation. In complex, heterogeneous biological media, no single spectral band corresponds exclusively to a specific functional vibration [25,26,27]. Alongside chemometric models, there is also interest in identifying a limited set of physicochemical parameters that offer clear interpretation.

Direct comparison of absolute Raman band intensities in milk is complicated by the system’s polydispersity and optical heterogeneity. Dispersed-phase particle size affects signal loss [28], while the spectral shape depends on autofluorescence, the baseline correction method, and the local composition of the sampled volume [29,30]. Scattering correction methods mitigate some optical distortions [31] but do not eliminate the influence of focusing and sample heterogeneity. Under these conditions, ratiometric analysis enables the comparison of responses from specific spectral regions within a single spectrum. The general principle of using Raman signal ratios was demonstrated by [32]; however, for milk emulsions, the selection of informative band pairs and the determination of their applicability limits require independent justification.

Regions associated with the major milk macro-components can be selected as potentially informative. While the Amide I and Amide III bands characterize peptide backbone vibrations, yet their profiles depend on secondary structure and overlapping contributions from other components [14]. The region near 1004 cm^−1^ is primarily associated with the breathing vibration of the phenylalanine aromatic ring; the characteristics of this vibration can vary depending on the conformation and microenvironment of the amino acid residue [33,34]. The signal near 1070 cm^−1^ includes contributions from lactose pyranose ring vibrations, while the region near 1440 cm^−1^ reflects C–H deformation vibrations, which are prominent in the lipid fraction [35,36]. The spatial heterogeneity in the distribution of milk components revealed by Raman mapping [37] precludes treating any of these bands as an absolutely stable internal standard without prior verification. Consequently, it remains insufficiently understood whether the ratiometric differences established during spray drying persist after rehydration and whether they allow for the differentiation of raw, pasteurized, and reconstituted milk derived from the same initial batch. The study hypothesized that the combined effect of spray drying and subsequent rehydration alters the relative Raman response of the protein, carbohydrate, and lipid fractions of the milk system, with some of the resulting differences persisting in the reconstituted milk and being detectable via ratiometric indicators.

The aim of the study was to identify ratiometric features in Raman spectra which are potentially applicable for the preliminary differentiation of raw, pasteurized, and reconstituted milk, as well as to evaluate differences between reconstituted samples prepared from whole milk powder obtained under two different spray-drying regimes.

## 2. Results

### 2.1. Raman Fingerprints and Structural Indices

#### 2.1.1. Fingerprint Identification and Gaussian Deconvolution

An overview of the obtained normalized Raman scattering spectra (wavenumber range 300–4500 cm^−1^) is presented in Figure 1.

The normalized Raman scattering profiles of the investigated milk samples maintained a baseline sequence of characteristic bands, though their relative shares were dynamically shifted by the food processing history (Figure 1). The most significant changes were identified within the multi-component spectral fingerprint domain (1000–1160 cm^−1^), where the intense stretching vibrations of the carbohydrate components overlay with functional protein and polar lipid bands (Figure 2).

To overcome the optical limits of raw intensity variations and ensure reproducible extraction of individual peak areas, a multi-peak Gaussian deconvolution procedure was applied. Four highly significant analytical bands (*p* < 0.001) appeared within the designated target window:

- A band near ~1005 cm^−1^, which is predominantly assigned to the symmetric ring-breathing vibration of the aromatic phenylalanine ring within the protein structure;
- A band near ~1063 cm^−1^, capturing the combined ν(C–O)_ring_ and ν(C–C) skeletal stretching of carbohydrates;
- A band near ~1083 cm^−1^, representing the skeletal stretching vibration of carbohydrate rings;
- A band near ~1121 cm^−1^, directly associated with the glycosidic bridge connectivity.

The extracted band center positions (x_c_) and the mathematically fitted integral areas (A) for all experimental milk processing groups are structured and presented in Table 1.

#### 2.1.2. Carbohydrate and Protein Index Processing Trends

Structural indices were calculated based on the verified analytical areas (A) (Table 2). The confidence interval (CI) for the structural index was calculated using the law of error propagation for indirect measurements.

95% confidence interval (Δ): for z = 1.96: Δ = 1.96 × SE_I_

1. Carbohydrate index: Lactose degradation/connectivity index (*I_lac_*)

It indicates the integrity of lactose glycosidic bonds [38,39].

(1) $$I_{\mathrm{lac}}=\frac{A_{1121}}{A_{1083}},$$

2. Protein index: Protein component stability index (*I_prot_*)

(2) $$I_{\mathrm{prot}}=\frac{A_{1005}}{A_{1063}},$$

This index utilizes the 1005 cm^−1^ band (associated with vibrations of the aromatic ring of phenylalanine—one of the key amino acids in milk proteins, specifically casein and whey fractions) and normalizes it against the 1063 cm^−1^ peak [34].

### 2.2. Multidimensional Projections and Binary Modeling

To identify latent patterns in the thermal degradation of dairy systems, the original indices (*I_lac_, I_prot_*) were transformed into orthogonal factors using the following mathematical algorithm (Table 3 and Table 4).

As indicated by the pairwise correlation matrix (Table 3), the initial ratiometric indicators exhibited statistical independence (r = 0.095), suggesting they already function as mutually orthogonal axes. Consequently, the subsequent Principal Component Analysis (PCA) was employed not for data dimensionality reduction or feature compression, but strictly as a standardized coordinate rotation operator. This transformation projects the independent carbohydrate and protein variance onto a normalized score plot space, thereby establishing a visually interpretable geometric framework for cluster boundary evaluation.

System of linear equations relating principal components to structural indices:

(3) $$\left\{\begin{array}{c} {\mathrm{PC}}_{1}=0.707\cdot I_{\mathrm{lac}}+0.707\cdot I_{\mathrm{prot}}\\ {\mathrm{PC}}_{2}=0.707\cdot I_{\mathrm{lac}}-0.707\cdot I_{\mathrm{prot}} \end{array}\;\right.,$$

The resulting mathematical model (Figure 3a) demonstrates that the carbohydrate and protein indices are independent.

PC axis 1 represents an integral indicator of the total processing load applied to the sample. Since the loading coefficients for both indices share the same sign and magnitude (+0.707 for both the protein and carbohydrate indices), movement from left to right along PC axis 1 reflects the simultaneous accumulation of changes in lactose and milk proteins. All non-dried liquid samples (raw milk H; pasteurized samples T-76, T-88, and T-98) are clustered strictly in the left half-plane (negative PC 1 values), as they did not undergo dehydration. Reconstituted dry samples (B-70 and B-95) shift to the right along PC axis 1, capturing structural changes resulting from drying.

PC axis 2 serves as a differential indicator of the processing regime. In the equation for PC 2, the carbohydrate index carries a positive sign (+0.707), while the protein index carries a negative sign (−0.707). This means PC axis 2 acts as a contrast marker, revealing which specific macro-component was more severely affected: an upward shift (positive values) indicates the dominance of changes in the carbohydrate phase, whereas a downward shift (negative values) indicates the dominance of protein phase denaturation. Regarding practical separation, PC axis 2 distinguishes between two drying classes: sample B-70 (low-temperature drying) shifts upward, reflecting lactose fluctuations alongside relatively mild protein damage, while sample B-95 (harsh high-temperature drying) shifts downward, signaling alterations in the protein pool that mathematically outweigh the changes in carbohydrates.

In addition to the above, binary logistic regression was used to differentiate between pasteurized and reconstituted milk based on the derived indices (Figure 3b).

For the standard log-odds formulation (Equation (6)), the logit coefficients were uniformly scaled by a factor of two relative to the linear discriminant boundary (Equation (5)). This linear scaling preserves the underlying geometric separation while maintaining integer-based coefficients for enhanced computational and algebraic clarity within the predictive framework.

Position of points on the graph:

Pasteurized milk zone (to the left/below the line, Z < 0): Characterized by a controlled upward increase in the protein index at low to moderate carbohydrate index values. Samples T-76, T-88, and T-98 align to form a vertical “temperature trend.”

Reconstituted milk zone (to the right/above the line, Z > 0): Reconstituted samples exhibit an upward shift. Point B-70 is displaced far to the right due to changes in lactose, while B-95 shifts upward due to denaturation of the protein phase.

The constructed geometric separation model demonstrates the topological isolation of the clusters. The separating line, described by Equation (4), forms an analytical barrier.

Z = 5.0 · *I_lac_* + 4.0 · *I_prot_* − 6.0, (4)

The pasteurized milk cluster (including raw milk H) is localized in the left half-plane, exhibiting a linear isochoric drift along the *I_prot_* axis that reflects the denaturation of the whey protein pool at 76–98 °C. Reconstituted milk samples (B-70, B-95) shift into the upper zone as a result of dehydration processes. The spatial separation of the experimental points attests to the discriminative power of the markers used and their potential application in automated chemometric quality control for food systems.

Logistic function for calculating the probability (P) of the presence of milk powder:

(5) $$P\left(\mathrm{reconstituted}\right)=\frac{1}{1+e^{-Y}},$$

(6) $$P=\frac{1}{1+e^{-\left(10.0\cdot I_{\mathrm{lac}}+8.0\cdot I_{\mathrm{prot}}-12.0\right)}},$$

where the logarithm of odds (Y) is

*Y* = 10.0·*I_lac_* + 8.0·*I_prot_* − 12.0, (7)

Mathematical verification of the model:

T-76: P = 0.004 (0.4% natural).

H (control): P = 0.029 (2.9% natural).

T-88: P = 0.029 (2.9%, natural).

T-98: P = 0.032 (3.2%, natural).

B-70: P = 0.968 (96.8%, reconstituted).

B-95: P = 0.999 (99.9%, reconstituted).

## 3. Discussion

### 3.1. Interpretation of Ratiometric Raman Indices

In the vibrational spectroscopy of milk matrices, the 1000–1160 cm^−1^ region is an area of spectral overlap. This region is dominated by intense ν(C–O) and ν(C–C) stretching vibrations of the lactose carbohydrate ring, which overlap with signals from polar lipids (phospholipids, sphingomyelin)—which constitute the milk fat globule membrane (MFGM)—specifically the ν_s_(PO_2_^−^) stretching vibrations of phosphate groups and the ν(C–O) stretching vibrations of ester groups [25,39]. Both bands (1121 and 1083 cm^−1^) fall within the region of C–O and C–C stretching vibrations associated with the pyranose ring and glycosidic linkages of lactose. Changes in their ratio presumably reflect alterations in the crystalline form of lactose (amorphous ↔ crystalline) or its binding to proteins [38,39].

Therefore, the ratiometric carbohydrate index employed here (*I_lac_* = *A*_1121_/*A*_1083_) can be termed the lactose degradation/binding index (*I_lac_*), as it reflects the integrity of lactose glycosidic bonds. The index value varies not only due to the thermal degradation of lactose but also as a result of changes in the physical state of the lipid pool [25].

For raw milk, the index value is 0.493. Upon heating (T-76, T-88, T-98), the value decreases to the range of 0.342–0.404; this can be attributed to the progression of various stages of the Maillard reaction (the interaction of lactose with protein amino groups during heating), which alters the local environment of lactose molecules [3,40]. The trend within the group is non-linear, potentially reflecting the complex equilibrium of lactose interconversion between forms (α- and β-anomers) in solution [10]. In the low-temperature drying sample (B-70), the index rises to 0.759. During gentle drying (≤70 °C), according to our working hypothesis, lactose transitions into a glassy (amorphous) state without reacting with proteins as intensely as it does at high temperatures [26]. Amorphous lactose produces a specific distribution of intensities for these bands. With high-temperature drying (B-95), the index drops to 0.383. It can be hypothesized that drying at ~95 °C triggers more profound changes: the amount of free lactose decreases as it binds to denatured proteins [17], bringing the index back to values similar to those observed in high-temperature pasteurization (T-98).

Protein component stability index (*I_prot_*). This index utilizes the 1005 cm^−1^ band (associated with vibrations of the aromatic ring of phenylalanine—a key amino acid in milk proteins, including casein and whey fractions) and normalizes it against the 1063 cm^−1^ peak. The frequency corresponding to the skeletal stretching vibrations of the C–O bond in the lactose pyranose ring was selected as the reference comparison band; this aligns with classic studies on dairy product spectroscopy [19,20]. Additionally, the 1063 cm^−1^ band is associated with C–N and C–C vibrations of the protein backbone or with lipid components.

Compared to raw milk (0.422), heating leads to an increase in the index. During pasteurization (T-76 → T-88 → T-98), a linear, dose-dependent increase in the protein index is observed, rising from 0.376 to 0.675. Reconstituted milk (B-70 → B-95) shows a sharp increase in the index. With low-temperature drying (B-70), the index rises to 0.978 (2.3 times higher than the control). With high-temperature drying (B-95), the index reaches a value of 1.840 (4.3 times higher than the control).

At temperatures of 88 °C (0.558) and especially 98 °C (0.675), intense thermal denaturation of whey proteins (primarily β-lactoglobulin) occurs [9,17]. Upon denaturation, the protein globule unfolds, exposing previously hidden phenylalanine residues, which increases the intensity of the 1005 cm^−1^ band. The slight decrease in the index at 76 °C (0.376) is likely attributable to the initial stage of protein aggregation occurring without complete unfolding of the globules [41] or to measurement error (±0.106).

The phenylalanine vibration at 1005 cm^−1^ is sensitive to the microenvironment [42]. Upon protein denaturation, casein micelles aggregate, shifting phenylalanine aromatic rings from a hydrophilic microenvironment into the hydrophobic cores of the aggregates. According to Almeida et al. [19], the size of casein micelles and fat globules directly determines the penetration depth of the 532 nm laser beam and the proportion of elastic (Rayleigh) scattering. Changes in the system’s dispersion state distort the baselines and relative intensities of protein and carbohydrate bands [13,43]. This causes a physical shift in the maximum of the ~1005 cm^−1^ band (by up to several cm^−1^) as well as a change in the peak’s full width at half maximum.

It should be noted that the normalization procedure based on the integral area of the entire spectrum (330–4050 cm^−1^) leads to a known chemometric effect—a redistribution of intensity fractions—whereby protein degradation in severely dried samples (B-95) and the decline of the 1005 cm^−1^ peak mathematically inflate the relative intensity of the lactose band [44]. However, in the context of the study’s objective—identifying the technological status of the raw material—this effect does not constitute a methodological flaw. Since the study does not aim to determine the absolute mass fraction of lactose, this feature serves as a cumulative diagnostic criterion. It mathematically exaggerates and amplifies the spectral differences between non-reconstituted and reconstituted systems, increasing the geometric distance between clusters in principal component space and enhancing the discriminative power of the binary logistic regression model [15,30,45].

The mathematical synergy between the actual chemical change and the accompanying normalization artifact acts as a non-linear stretching operator on the feature space. This increases inter-class variance (the spread between the groups of non-reconstituted and reconstituted systems) along the diagnostic axis (PC1). In chemometrics and pattern recognition tasks, this maximizes the geometric distance (Mahalanobis or Euclidean) between clusters, thereby reducing the probability of model false positives [29].

This phenomenon aligns with established chemometric principles, where total area normalization routinely triggers a competitive mathematical redistribution of spectral intensity shares among overlapping components. Rather than introducing a methodological flaw, this artifact functions as a non-linear stretching operator that amplifies subtle biochemical divergences. By artificially expanding the Mahalanobis and Euclidean distances between the non-reconstituted and reconstituted clusters in the latent feature space, the preprocessing routine significantly suppresses model false-positives and sharpens the class-separation threshold.

Analysis of the orthogonal decomposition parameters of the standardized correlation matrix (Figure 3a) demonstrated an effective distribution of feature information between the two identified principal components. The resulting mathematical model confirms that the carbohydrate and protein indices are independent.

Multivariate optimization of the separating hyperplane (Figure 3b) yielded a linear discriminant equation featuring a geometric class separation margin (Equation (4)).

The geometric solution to this equation, expressed in the physical axes of the indices under study, is described by the linear function *I_prot_* = 1.50 − 1.25 · *I_lac_*. This analytical threshold ensures separation: the pool of non-reconstituted samples (raw control, samples T-76, T-88, T-98) is localized below the line in the negative value range (Z ≤ −1.71), with no overlap with the data points’ error intervals. Reconstituted milk samples (B-70, B-95) are isolated in the upper half-plane (Z ≥ +1.71).

Based on the obtained coefficients, a logit model was formulated with the log-odds (Equation (7)). Calculation of prior probabilities showed that for all types of non-reconstituted milk, the false-positive probability does not exceed 3.2%, while the model-predicted probability for reconstituted milk ranges from 96.8% to 99.9%, confirming the potential of this screening method.

Establishing the system’s unified physicochemical and biological basis enabled the high-precision verification of the technological trend regarding protein and lactose degradation. This approach aligns with standard practices in developing new physicochemical analysis methods [19,46], wherein the fundamental viability of a chemometric pattern recognition model is first demonstrated using a controlled model series prior to subsequent testing on large-scale industrial samples.

Despite the high discriminative performance achieved by the binary logistic regression model, certain mathematical and methodological limitations must be acknowledged. First, because the classification model was developed and evaluated using the same experimental dataset derived from a single homogeneous batch of raw milk, the reported high classification accuracies (96.8–99.9%) represent resubstitution (training) metrics rather than independent validation performance. In chemometrics, this can lead to overfitting, meaning that the established log-odds coefficients (Equation (7)). are highly tailored to this specific matrix composition. Furthermore, the geometric distribution of the samples exhibited complete separation in the two-dimensional feature space, which is known to cause instability in maximum likelihood estimation, potentially inflating the standard errors of the regression coefficients. Finally, because the dataset dimensionality was not compressed (retaining two principal components for two initial ratiometric indices), the PCA step served primarily as a coordinate rotation to demonstrate feature orthogonality rather than a true reduction of independent variables. Therefore, the current regression model should be considered a preliminary proof-of-concept. To ensure the transferability and robustness of these spectroscopic calibrations against biological, seasonal, and instrumental drift, further validation utilizing penalized likelihood methods (e.g., Firth’s regression) on large-scale, independent industrial batches is strictly required. The data in Table 5 indicate the potential for further research in the stated direction, presenting a comparison of the technical and economic characteristics of existing alternative methods.

The most accurate and selective technique is High-Performance Liquid Chromatography (HPLC), regulated by the ISO 18329 standard. This method directly targets furosine and demonstrates superior sensitivity, with a limit of detection (LOD) from 0.50 mg/100 g (5 ppm) till 0.02 ppm. The method exhibits good reproducibility, with a relative standard deviation (RSD) ranging from 3.1% to 10.1%, and a high recovery rate (Recovery: 79.9–94%), making it the gold standard for reference analysis [49,50].

As an alternative, the colorimetric method is utilized to determine hydroxymethylfurfural (HMF) [47]. However, its metrological performance is inferior to that of chromatography. But, according to modern literature data [49], the LOD for HMF could be 0.05 ppm, while the recovery rate ranges between 73% and 91% with a variance (RSD) of 3.8–12.50%. The primary systemic drawback of colorimetry is the interference caused by the native sugar background of the milk, which can lead to overestimations of the analyte. Colorimetry quantifies exclusively free HMF released into the whey, leaving protein-bound HMF undetected unless additional harsh cleavage steps are introduced. The proposed RS method analyzes the native structure of both proteins and carbohydrates in an intact, non-destructive state, providing an integrated assessment of the total thermal footprint.

Fourier-Transform Infrared (FTIR) Spectroscopy, which complies with ISO 9622/IDF 141 [48] standards, provides a rapid, indirect estimation of macro-component bond vibrations. Its accuracy relies heavily on proper instrument calibration, yielding a root-mean-square error of prediction (RMSEP) of approximately 5–10%. Spectral methods do not measure the concentration of markers directly; instead, they evaluate the indirect heat load on the milk matrix (including lactulose and furosine levels) via mathematical modeling.

The colorimetric method for HMF [47] determination requires trichloroacetic acid (TCA). TCA—a highly corrosive agent demanding specialized disposal procedures—and thiobarbituric acid. Similarly, HPLC requires toxic acetonitrile and concentrated hydrochloric acid. In contrast, the proposed Raman spectroscopy (RS) method complies fully with Green Chemistry principles, as it generates zero chemical waste.

### 3.2. Limitations and Future Validation Requirements

Although the developed Raman ratiometric approach, combined with the binary logistic regression model, demonstrated a capability to differentiate non-dehydrated dairy matrices from reconstituted milk, several critical limitations must be explicitly acknowledged to define the boundaries of the method’s current applicability.

As a preliminary proof-of-concept study, the primary limitation stems from the sample matrix design. The entire experimental trial was executed using a single homogeneous batch of raw bovine milk sourced from a single commercial facility. Consequently, the reported high classification probabilities (96.8–99.9%) represent resubstitution performance within a tightly controlled training dataset rather than an independent validation. In real-world food authentication scenarios, the structural and chemical profiles of dairy systems are subject to significant biological and technological variability. To transition this method from a laboratory prototype to a robust process analytical technology (PAT), the proposed calibrations must undergo rigorous multi-batch validation. We have planned further research with a systematic assessment of the impact of the following variables.

Biological and seasonal dynamics. Variations in dairy composition driven by seasonal fluctuations, animal genetics (breed specificity), lactation stages, and regional cattle feed regimes can fundamentally alter the baseline concentrations of native proteins, free/bound lactose ratios, and mineral balances.

Influence of matrix fat pool. The spectral domain evaluated (1000–1160 cm^−1^) is an area of complex biochemical overlap. Although the selected bands primarily track carbohydrate and protein backbones, fluctuations in the fat fraction (e.g., skimmed vs. whole milk) and the technological state of the milk fat globule membrane (MFGM) can induce shifts in baseline scattering. Changes in the polydispersity and size distribution of fat globules directly modulate the penetration depth of the 532 nm laser and the ratio of elastic Rayleigh scattering, which may distort the relative peak areas of *A*_1005_ and *A*_1063_.

Instrumental and Environmental Drift: The reproducibility of the ratiometric thresholds *I_lac_* and *I_prot_* must be verified across different commercial Raman spectrometers to assess the robustness of the automated Gaussian deconvolution script against variations in detector sensitivity, laser power fluctuations, and optical focusing tolerances.

Furthermore, the mechanistic explanations proposed for the carbohydrate index *I_lac_* modifications—associating them with lactose amorphous crystallization during low-temperature drying or protein binding during harsh thermal stress—remain structural hypotheses. To definitively corroborate these biochemical transitions, future work must incorporate complementary orthogonal validation methods. This includes utilizing High-Performance Liquid Chromatography (HPLC) to accurately quantify furosine and lactulose progression, alongside X-ray Diffraction (XRD) to track the absolute crystalline-to-amorphous transformations of lactose within the dried matrices.

Finally, from a regulatory and practical inspection perspective, a critical step involves establishing the exact Limit of Detection (LOD) for reconstituted milk adulteration. The current binary model is calibrated for “all-or-nothing” technological states (100% liquid vs. 100% reconstituted matrices). In commercial fraud, reconstituted powder is frequently blended with fresh milk at lower ratios. Establishing a robust quantification threshold requires immediate “spiking experiments” involving blind-coded binary mixtures at varying contamination levels (e.g., 5%, 10%, 20%, and 50% replacement). Comprehensive multi-center validation adhering to ISO 5725 [51] guidelines is strictly required before this Raman screening method can be adopted for official food quality control systems and regulatory enforcement.

## 4. Materials and Methods

### 4.1. Research Objects and Sample Matrix Preparation

The baseline object investigated in this study was raw whole bovine milk obtained from a single homogeneous batch at the reception unit of the “Italianskie Traditsii” commercial cheesemaking facility (Moscow, Russia). To systematically isolate the effects of industrial engineering pathways on nutrient structure, six distinct experimental treatment groups were prepared from this identical source matrix:

H: Raw whole bovine milk without any additional thermal or physical processing (used as the control).

T-76: Pasteurized liquid milk processed at a target temperature of 76 °C with a continuous holding time of 20 min.

T-88: Pasteurized liquid milk processed at a target temperature of 88 °C with a continuous holding time of 5 min.

T-98: Pasteurized liquid milk processed at a target temperature of 98 °C with a continuous holding time of 5 min.

B-70: Reconstituted liquid milk prepared from whole milk powder obtained via pilot-scale spray drying maintaining an outlet air temperature ≤70 °C.

B-95: Reconstituted liquid milk prepared from whole milk powder obtained via pilot-scale spray drying maintaining a temperature outlet air regime of ~95 °C.

### 4.2. Experimental Workflow and Chronology

The full experimental trial was executed over two consecutive days to minimize temporal biological degradation of the matrix. On the first day, raw milk aliquots were sampled from the baseline reception batch and immediately subjected to thermal processing at (76, 88, and 98) °C. Raman spectra of the fresh raw control (H) and the three pasteurized fluids (T-76, T-88, T-98) were registered immediately following heat treatment and sample cooling.

Concurrently, a separate 40 kg volume of the identical baseline raw milk batch was dispatched to a pilot dehydration facility. On the second day, the resulting whole milk powder variants were transferred to the P.N. Lebedev Physical Institute (LPI, Moscow, Russia) for fluid rehydration and immediate subsequent spectroscopic analysis.

### 4.3. Industrial Processing Protocols

#### 4.3.1. Thermal Processing

Thermal pasteurization was performed utilizing an XT5018-GP38 digital water bath featuring gravity convection (Xutemp Temptech Co., Ltd., Hangzhou, China). The real-time internal temperature of the milk matrix was continuously monitored via a calibrated immersion digital thermometer. The continuous holding countdown was initiated strictly after the center of the liquid matrix reached the designated target temperature. Three distinct thermal regimes were evaluated: 76 °C for 20 min, 88 °C for 5 min, and 98 °C for 5 min. Upon completion of the designated holding window, all samples were immediately forcibly cooled in a circulation ice water bath to a stable room temperature of 22 ± 1 °C to avoid thermally induced transformations.

#### 4.3.2. Spray-Drying Dehydration

Whole milk powder samples were generated utilizing a customized pilot-scale spray drying tower at the All-Russian Research Institute of the Dairy Industry (VNIMI, Moscow, Russia) operating at a baseline evaporation capacity of 20 kg/h of moisture. Drying was carried out using two contrasting thermal regimes—low-temperature and high-temperature—achieved by modulating the “thermal budget” during the spray-dehydration stage. The raw whole milk was heated to a temperature of 65–70 °C. Preliminary concentration was performed in a vacuum evaporator at a boiling point of 70 °C until an intermediate solids content of 25.0 ± 2% was reached. At an inlet temperature of 65–70 °C, the concentrated product was fed into a pneumatic nozzle at a flow rate of 25–28 L/h. For the B-70 sample, the heating of the gaseous drying medium (purified atmospheric air) at the inlet to the drying chamber was in the range of 155–165 °C. The temperature of the exhaust air at the tower outlet was maintained between 65 °C and 68 °C. Final drying and cooling of the product were carried out using dehumidified air at a temperature of 20–22 °C. For the B-95 sample, the drying medium temperature at the inlet was 185–200 °C, while the exhaust air temperature at the outlet was 90–95 °C. Thus, the outlet drying air temperature was strictly kept at ≤70°C and ~95 °C, respectively, under a constant atomization pressure.

#### 4.3.3. Reconstitution

The spray-dried whole milk powder samples were reconstituted with ultra-pure water to achieve a strictly calculated total solids mass fraction of 12.1%, matching the native control. A precise powder mass of 12.6 g was mixed with 87.4 g of ultrapure water (Type II analytical grade) preheated to 45 °C. The mixture was held inside a stabilized water bath at 45 °C for 30 min to guarantee complete thermodynamic hydration of the nutrients.

Subsequently, the matrix was thoroughly homogenized utilizing a DLAB MX-S vortex mixer (DLAB Scientific Co., Beijing, China) operating at 1500 rpm for exactly 5 min. The reconstituted fluid samples were then forcibly cooled and dynamically stabilized at 22 ± 1 °C inside a KT 53 Peltier-cooled analytical incubator (Binder, Tuttlingen, Germany) prior to optical scanning.

### 4.4. Raman Spectroscopic Instrumentation and Settings

Raman scattering spectra were recorded using a high-resolution M532 Raman microscope system composed of an EnSpectr R532^®^ express analyzer fiber-coupled with a professional U300M confocal optical microscope (OOO Spectr-M, Chernogolovka, Russia). The instrument was equipped with a continuous-wave solid-state laser emitting at a strict excitation wavelength of 532 nm and featured an automated internal wavelength calibration routine based on a silicon reference standard.

The backscattered signal was collected through a long-working-distance 50× planar objective lens possessing a numerical aperture (NA) of 0.75. The absolute laser power arriving at the sample surface was strictly capped at ≤50 mW to prevent localized thermal degradation or micro-boiling of the fluid matrix. Spectra were acquired across a continuous wavenumber range of 160–4200 cm^−1^ with a stable spectral resolution maintained between 4 and 6 cm^−1^. The system utilized a reflective diffraction grating with a holographic segment of 1800 lines/mm and a 20 μm confocal pinhole, yielding a laser spot focal diameter of ~5 μm. Each single saved spectrum was generated by averaging 10 sequential accumulations with an exposure time of 5 s per acquisition (total integration time of 50 s per sample). All physical measurements were conducted inside a temperature-stabilized dark laboratory room at 22 ± 1°.

### 4.5. Spectral Acquisition Window and Scanning Routine

Before starting measurements, the stability of the instrument’s wavenumber scale was verified using an external crystalline silicon standard. If the position of the characteristic peak deviated from the theoretical value (520.5 cm^−1^) by more than ±1 cm^−1^, an automatic calibration of the Raman shift axis was performed using the EnSpectr PRO software (Scientific Edition).

Immediately prior to spectral recording, each liquid sample was homogenized via vortexing for 1 min at 1500 rpm. A 50 μL aliquot of the liquid milk was deposited via a high-precision micropipette onto a clean glass cover slide (0.17 mm thickness). The spatial position of the liquid–air interface was precisely locked by maximizing the specular reflection intensity of the integrated guide laser beam. Using a motorized piezo-driven Z-axis nanostage, the laser focal volume was then translated down to a fixed depth of exactly 100 μm beneath the surface into the bulk liquid phase to avoid surface evaporation artifacts. Microscopic observation confirmed the occurrence of localized, laser-induced convective micro-stirring within the sampling volume during data acquisition. Crucially, this thermal convection did not destabilize the baseline or alter the focal plane registration; rather, it served as a dynamic homogenization mechanism. This effect actively assisted in averaging out the spatial heterogeneity governed by localized clusters of fat globules and casein micelles, thereby enhancing the reproducibility of the captured ensemble spectra. For each experimental sample treatment group, a series of 10 independent measurements were conducted. The glass cover slides were discarded and replaced after testing each individual sample.

### 4.6. Spectral Data Preprocessing and Chemometrics

#### 4.6.1. Preliminary Mathematical Processing

Raw Raman spectra were mathematically processed using OriginPro 2021b (v9.85) software (OriginLab Corporation, Northampton, MA, USA) using a uniform scripting sequence. Cosmic ray anomalies, erratic electronic spikes, and random noise were automatically eliminated via the integrated EnSpectr R532^®^ control software. Spectral smoothing was performed using the Savitzky–Golay algorithm with a frame window size of 7 points and a 2nd-order polynomial.

To eliminate the intensive non-linear matrix autofluorescence background, a baseline correction routine was applied using the Asymmetric Least Squares (ALS) smoothing method. Following baseline subtraction, area normalization was executed over an integrated wavenumber window of 330–4050 cm^−1^. Normalization was achieved by dividing the absolute scattering intensity at each individual wavenumber point by the total integrated area calculated within the selected window, transforming the data into relative scattering shares.

#### 4.6.2. Multi-Peak Gaussian Deconvolution and Ratiometric Indices Calculation

To achieve clean numerical separation of overlapping vibrational bands and accurately resolve individual peak areas within the congested structural fingerprint domain 1000–1160 cm^−1^, curve fitting was executed using a multi-peak Gaussian function. Four analytical bands tracking true peak maximum centers at ~1005, ~1063, ~1083, and ~1121 cm^−1^ were defined. The respective fitted integral areas were logged as *A*_1005_, *A*_1063_, *A*_1083_, and *A*_1121_. Deconvolution and true vertex tracking were executed independently for each replication spectrum, and the target ratiometric structural indices were computed based on the extracted area values.

#### 4.6.3. Statistical Processing and Multivariate Modeling

Quantitative data are presented as the arithmetic mean accompanied by its associated standard error interval (M ± SE), where SE denotes the standard error of the mean based on *n* = 10 technical replicates. Multivariate dimensionality reduction was performed via Principal Component Analysis (PCA) based on the standardized pairwise correlation matrix of the calculated structural indices (OriginLab). The mathematical separation lines of technological processing states were locked using a multivariate binary logistic regression model, with the confidence boundaries of the classification points calibrated for a 95% confidence interval (Z = 1.96). Instead of fitting a standard logistic regression independently from scratch, the model parameters were determined via a direct analytical mapping of an optimized Linear Discriminant Analysis (LDA) separating hyperplane. The objective function for the initial linear discriminant Z = w^T^x + *w*_0_ was tuned to maximize the geometric separation margin between the non-reconstituted and reconstituted sample pools, yielding the baseline equation (Equation (7)).

To transform this rigid geometric classifier into a probabilistic framework, the discriminant scores were calibrated into a logit model. Mathematically, for a two-class problem with equal covariance matrices and multivariate normal distributions, the log-odds (logit) function ln(p/(1 − p)) is directly proportional to the linear discriminant function Z. Rather than executing an iterative Maximum Likelihood Estimation (MLE) procedure—which can suffer from instability or non-convergence when classes are perfectly separated with zero overlap—the logit coefficients were determined via exact linear scaling of the LDA weight vector by a scaling factor of k = 2.0. This scaling factor analytically satisfies the maximum likelihood criterion under the assumed distributional constraints, ensuring that the geometric slope of the threshold line (Y = 1.50 − 1.25·X) remains perfectly preserved. The posterior probabilities of classification were then evaluated using the standard logistic sigmoid function. The robustness of the resulting parameters was validated by calculating prior and posterior probabilities.

### 4.7. Ethics, Large Datasets, and Institutional Approvals

Ethical Approval: This study did not involve human subjects, live animals, or tissue interventions requiring ethical approval. The raw bovine milk matrix evaluated was a standard commercial agricultural commodity collected from a food processing facility in strict accordance with industrial quality control standards. Therefore, no ethical approval codes or institutional authority clearances were required.

Large Datasets Depositories: The datasets generated during this study did not include large-scale omics, high-throughput sequencing, or genetic arrays that require deposition in public archival repositories (e.g., GenBank, NCBI, or PDB).

### 4.8. Data Availability Statement

The full raw and processed datasets, spectroscopic arrays, deconvolution protocols, and chemometric mathematical models generated during the current study are available from the corresponding author upon reasonable request.

### 4.9. Disclosure of Generative AI (GenAI) Usage

Generative artificial intelligence (GenAI) tools were not utilized for the study design, experimental data collection, spectroscopic measurements, statistical analysis, mathematical modeling, graphics generation, or scientific interpretation of the raw experimental results. GenAI (Google. Gemini, October 2026 version) was deployed exclusively for superficial text editing, grammar optimization, technical formatting alignment, and academic English translation of the pre-existing draft text to fulfill the submission standards of the journal.

## 5. Conclusions

In this study, Raman spectroscopy, combined with multi-peak Gaussian deconvolution and ratiometric analysis, was successfully demonstrated as a highly sensitive, non-destructive tool for monitoring structural and conformational alterations in dairy matrices. The proposed carbohydrate (*I_lac_*) and protein (*I_prot_*) indices, derived from the critical fingerprint region (1000–1160 cm^−1^), were shown to be mathematically independent markers that effectively capture phase transitions in lactose and the thermal unfolding of whey proteins. As a preliminary proof-of-concept, the multivariate binary logistic regression model established a clear analytical boundary, achieving complete geometric separation between the non-dehydrated dairy pool (raw control and pasteurized samples) and reconstituted liquid milk. Although the reported model-predicted probabilities (96.8–99.9%) are highly promising, they reflect internal training performance based on a single homogeneous milk batch. Consequently, while the mathematical synergy between actual chemical changes and spectral normalization artifacts amplifies inter-class variance, further validation across independent industrial batches is strictly required. Future research must evaluate the robustness of these ratiometric calibrations against seasonal biological fluctuations, matrix composition variability, and instrumental drift to ensure the scalability of the method for automated food quality control systems.

## Figures and Tables

**Figure 1:**
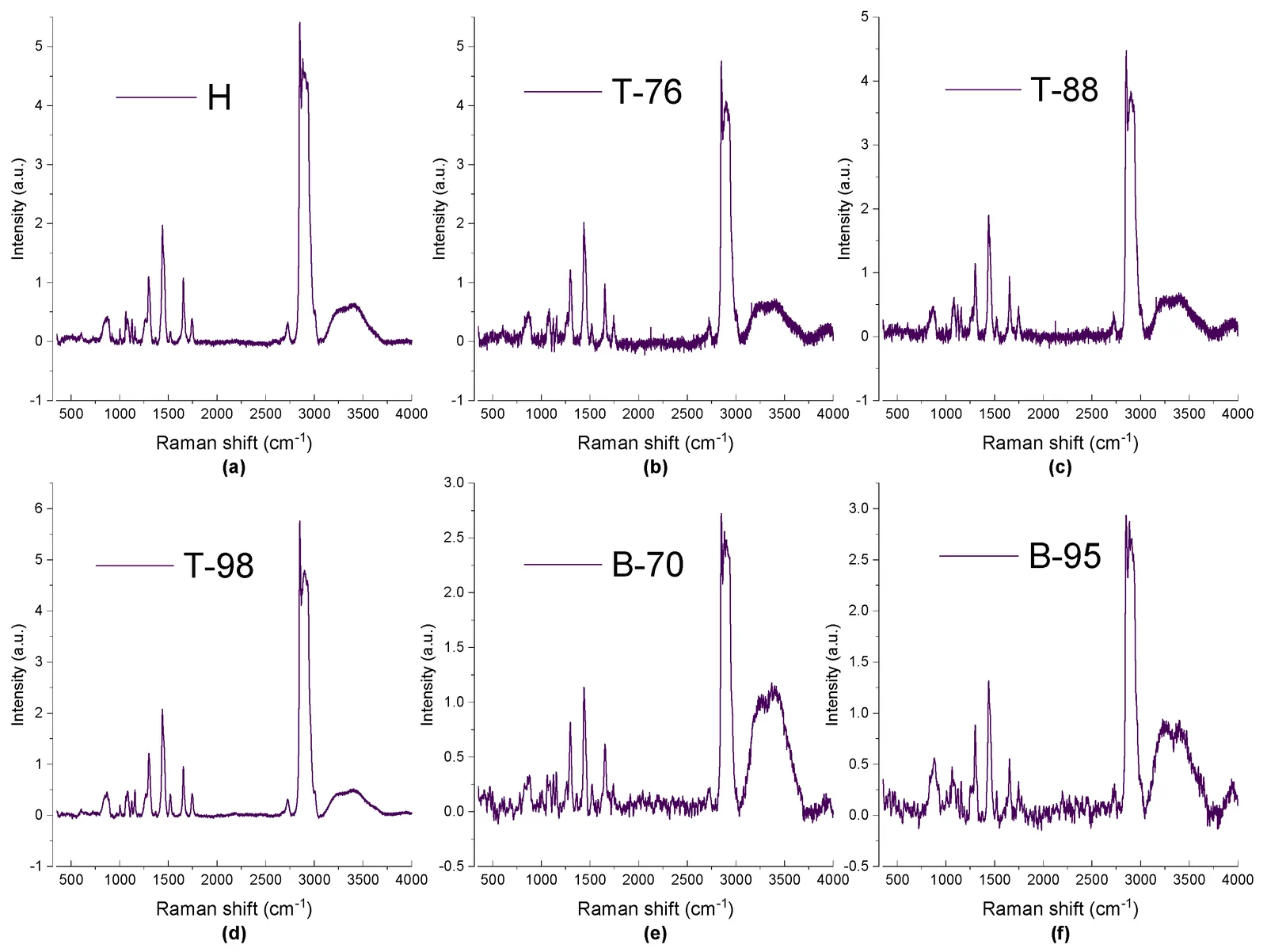
Overview of normalized Raman scattering spectra (wavenumber range 300–4500 cm^−1^) of whole bovine milk under different technological treatments: (**a**) raw control (H); (**b**–**d**) liquid milk pasteurized at 76 °C, 88 °C, and 98 °C, respectively; (**e**,**f**) reconstituted milk prepared from powders spray-dried at outlet air temperatures ≤70 °C (B-70) and ~95 °C (B-95).

**Figure 2:**
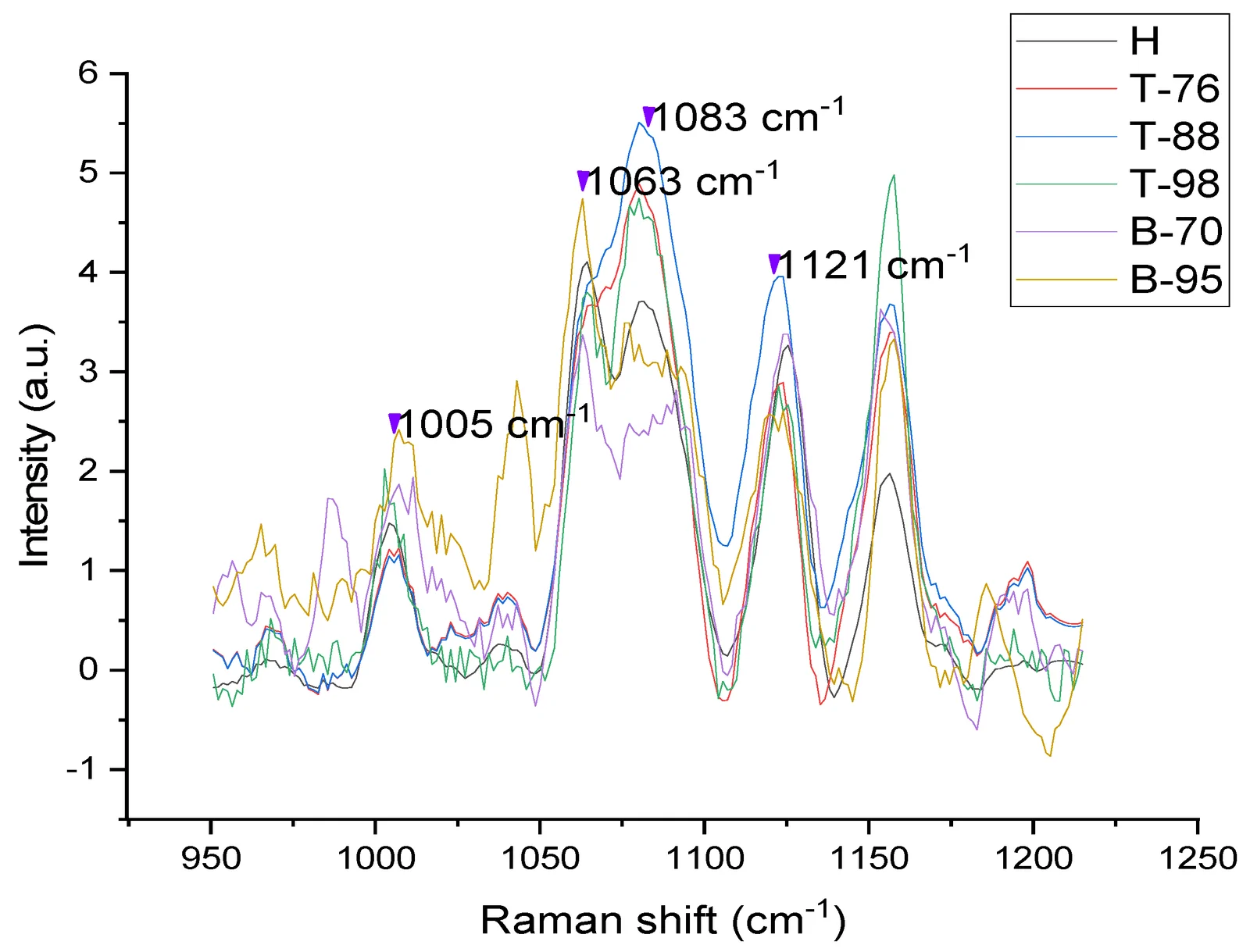
The Raman fingerprint region (950–1250 cm^−1^) showcasing the overlay of normalized spectra for all evaluated dairy matrices. Blue markers indicate the positions of the key analytical bands (~1005, ~1063, ~1083, and ~1121 cm^−1^) utilized for the construction of ratiometric structural indices.

**Figure 3:**
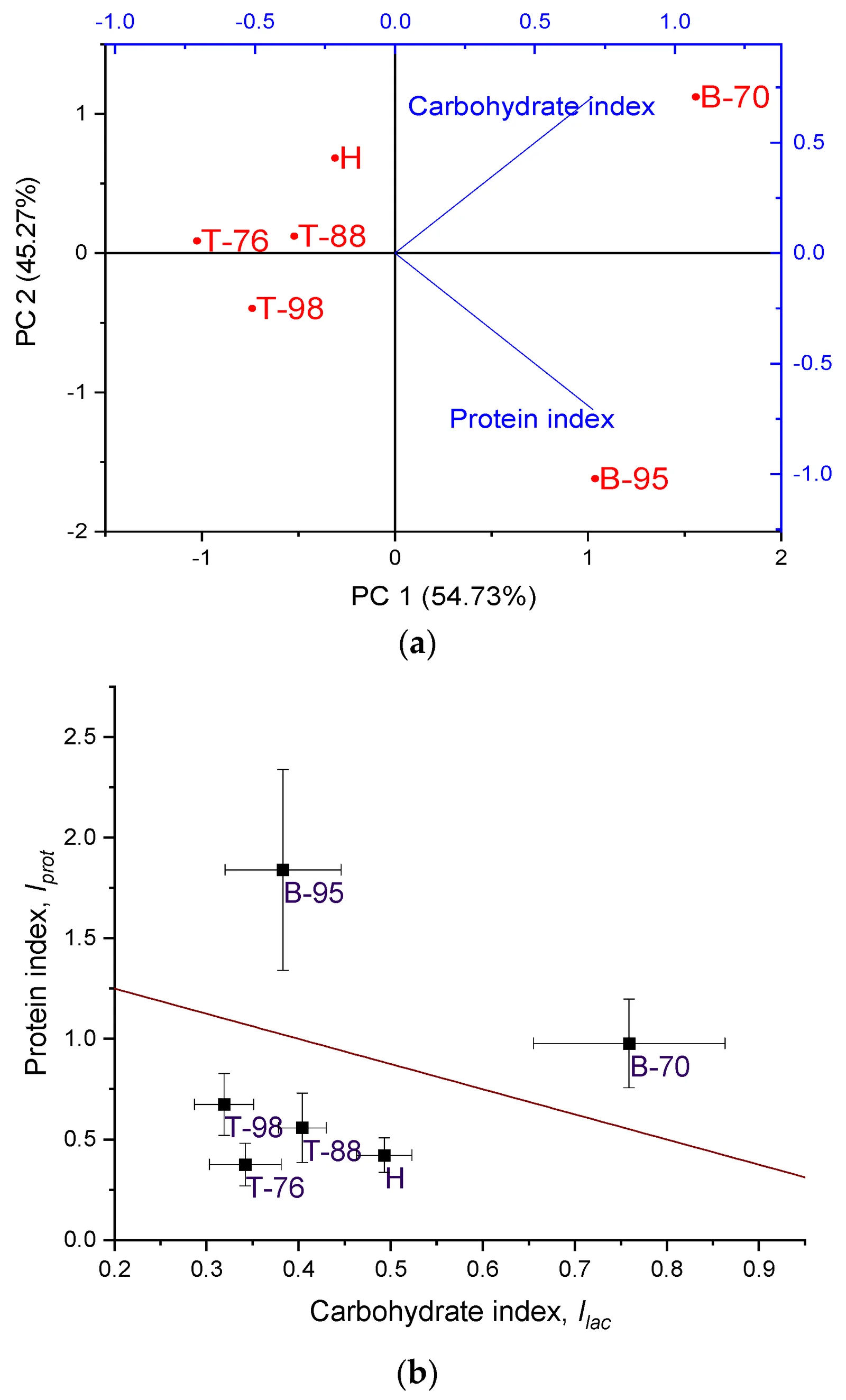
Multidimensional projections of dairy processing datasets. (**a**) Principal Component Analysis (PCA) score plot mapping integrated component load along the orthogonal PC1–PC2 axes. (**b**) Geometric model of binary logistic regression separation mapping the physical coordinate barrier.

**Table 1:** Deconvolution parameters of key analytical bands in Raman spectra (using the Gaussian method) for milk samples with different technological histories (M ± SE) ^1^.

Analytical Band. cm^−1^	Parameter/Variable	H	T-76	T-88	T-98	B-70	B-95
~1005	x_c_, cm^−1^	1005	1005.7	1005.8	1004.8	1008.5	1010.4
νPhe	±SE	0.24	0.46	0.38	0.37	0.71	0.66
(Phenylalanine)	A (×10^−3^)	1.88	1.59	1.30	1.93	3.94	8.76
	±SE (×10^−4^)	0.865	1.47	1.11	1.45	3.46	5.54
~1063	x_c_	1062.8	1063.3	1062.3	1063.2	1062.7	1061.2
ν(C-O)ring,	±SE	0.15	0.43	0.27	0.25	0.34	0.24
ν(C-C),	A (×10^−3^)	4.46	4.23	2.33	2.86	4.03	4.76
δ(C-O-H)	±SE (×10^−4^)	2.08	4.65	3.07	2.55	3.03	5.85
~1083	x_c_	1082.5	1081.7	1081.9	1081.7	1085.7	1083.3
ν(C-O),	±SE	0.29	0.43	0.3	0.3	0.55	0.79
ν(C-C),	A (×10^−2^)	0.99	1.16	1.70	1.17	0.723	1.42
δ(C-O-H)	±SE (×10^−4^)	2.48	5.02	4.18	3.38	4.05	8.72
~1121	x_c_	1124.5	1121.8	1121.5	1122.9	1124.8	1122.8
ν(C-O),	±SE	0.12	0.18	0.14	0.23	0.25	0.34
ν(C-C),	A (×10^−3^)	4.90	3.96	6.88	3.73	5.49	5.45
δ(C-O-H)	±SE (×10^−4^)	0.94	1.49	1.47	1.54	2.30	3.07

^1^ Data obtained from *n* = 10 independent technical replicates for each individual processing group. Note: x_c_—band center position. A—fitted band area. SE—standard error of measurement.

**Table 2:** Calculated ratiometric indices for milk macrocomponents under different food processing pathways (M ± Δ, 95% CI).

Sample Group	Technological Processing History/Status	Carbohydrate Index *I_lac_* (*A*_1121_/*A*_1083_)	Protein Index *I_prot_* (*A*_1005_/*A*_1063_)
H	Raw whole bovine milk (control)	0.493 ± 0.030	0.422 ± 0.086
T-76	Pasteurized liquid milk (76 °C. 20 min)	0.342 ± 0.039	0.376 ± 0.106
T-88	Pasteurized liquid milk (88 °C. 5 min)	0.404 ± 0.026	0.558 ± 0.172
T-98	Pasteurized liquid milk (98 °C. 5 min)	0.319 ± 0.032	0.675 ± 0.154
B-70	Reconstituted liquid milk (spray-dried ≤70 °C)	0.759 ± 0.104	0.978 ± 0.221
B-95	Reconstituted liquid milk (spray-dried ~95 °C)	0.383 ± 0.063	1.840 ± 0.499

**Table 3:** Pairwise correlation matrix of initial indices.

Index	*I_lac_*	*I_prot_*
*I_lac_*	1	0.095
*I_prot_*	0.095	1

**Table 4:** Statistical performance parameters of the two-component Principal Component Analysis (PCA) model.

	Eigenvalue (λ)	Explained Variance, %	% Cumulative Variance, %
PC1	1.095	54.73%	54.73%
PC2	0.905	45.27%	100.00%

**Table 5:** Comparison matrix of milk heat treatment control methods.

Analytical Method	Target Marker	Chemical Sample Preparation	Analysis Time (per 1 Test)	Reagent Cost (per 1 Test, USD)	Total Cost per Test (Incl. Labor, USD)	Capital Cost (Equipment, USD)
HPLC (*ISO 18329* [18])	Furosine, lactulose	Acid hydrolysis of proteins (6N HCl, 110 °C, 18–24 h), filtration, solid-phase extraction (SPE).	~20–25 h (*total*) ~90 min (*manual*)	10.00–35.00	25.00–55.00	30,000–80,000
Colorimetry [47]	Hydroxymethylfurfural (HMF)	Acid hydrolysis, protein precipitation (TCA), centrifugation, reaction with thiobarbituric acid (TBA), water bath incubation (40 °C, 40 min).	~60–85 min (*total*) ~25 min (*manual*)	1.50–3.00	15.00–25.00	1500–4000
IR Spectroscopy (*ISO 9622* [48])	Macro-component bond vibrations	Direct sample introduction. Automatic heating of the flow cell by the instrument.	~2–3 min (*automated*)	<0.20 (*rinsing buffers only*)	<0.60	10,000–65,000
Proposed RS Method (*Raman Chemometrics*)	*I_lac_* and *I_prot_* indices (*protein conformation, Maillard products*)	Rapid dispensing into a glass cuvette/vial without destroying the native milk structure.	~2–10 min (*if it will be automated*)	<0.10 (*no reagents required*)	<0.40	10,000–35,000

## Data Availability

The original contributions presented in this study are included in the article. Further inquiries can be directed to the corresponding author.
